# Concordance of an Artificial Intelligence Model (ChatGPT 4.0) with Physician Decisions in Smoking Cessation Clinics: A Comparative Evaluation

**DOI:** 10.3390/healthcare13182283

**Published:** 2025-09-12

**Authors:** Yagmur Gokseven Arda, Guzin Zeren Ozturk

**Affiliations:** Department of Family Medicine, Şişli Hamidiye Etfal Training and Research Hospital, Faculty of Medicine, University of Health Sciences, 34396 Istanbul, Türkiye; guzin.zerenozturk@sbu.edu.tr

**Keywords:** smoking cessation, artificial intelligence, decision support systems

## Abstract

**Background:** Smoking is one of the leading causes of preventable mortality worldwide. Smoking cessation treatments require personalized therapeutic approaches. Artificial intelligence (AI) is increasingly utilized in clinical decision support systems; however, its role in smoking cessation treatment remains underexplored. This study aims to evaluate the concordance between ChatGPT-4.0-generated treatment recommendations and physician decisions in smoking cessation therapy. **Methods:** This retrospective and descriptive study was conducted by reviewing the electronic records of patients who presented to a Smoking Cessation Clinic. The ChatGPT-4.0 model was used to compare AI-generated treatment recommendations with physician-prescribed therapies. Concordance rates and the quality of AI-generated information (inappropriate, useful, or perfect information) were assessed. Statistical analyses were performed using SPSS 25.0. **Results:** A total of 82 patient records were analyzed. The mean age was 40.71 ± 12.87 years (range: 19–69). The overall concordance rate between physicians and ChatGPT-4.0 was 67.1%. Regarding ChatGPT-4.0-generated information quality, 32.9% of cases received inappropriate recommendations, 36.6% received useful recommendations, and 30.5% received optimal recommendations. ChatGPT-4.0 provided inappropriate recommendations in 81.5% of cases involving chronic diseases and 77.8% of cases involving regular medication use (*p* = 0.021, *p* = 0.030, respectively). ChatGPT-4.0 achieved the highest rate of optimal recommendations (52.0%) for cytisine therapy. **Conclusions:** ChatGPT-4.0 can serve as a supportive tool in smoking cessation treatment. However, it remains insufficient in managing complex clinical cases, emphasizing the necessity of physician oversight in final decision-making. Enhancing AI models with larger and more diverse datasets may improve the accuracy of treatment recommendations.

## 1. Introduction

Smoking is one of the leading causes of preventable mortality worldwide and is recognized as a major risk factor for numerous chronic diseases. According to the World Health Organization (WHO), tobacco-related diseases cause over 8 million deaths annually [1]. This alarming statistic has made tobacco control a key priority in global health policies. Smoking cessation treatments provide effective interventions at both individual and societal levels to manage this addiction and mitigate the harmful effects of tobacco. However, as individuals respond differently to treatment, personalized therapeutic approaches are becoming increasingly important [2].

In recent years, artificial intelligence (AI) technologies have increasingly been integrated into healthcare systems, leading to transformative developments in clinical decision support. AI models can rapidly analyze complex and multidimensional data, offering tailored recommendations to support healthcare professionals in diagnostic and treatment processes [3]. In smoking cessation specifically, treatment success is influenced by numerous factors, including sociodemographic characteristics, nicotine dependence levels, comorbidities, and motivational status [4]. Given this complexity, decision-making tools capable of adapting to individual patient profiles may enhance clinical outcomes.

Despite the expanding role of AI in medicine, few studies have explored its potential in smoking cessation management. To address this gap, the present study evaluates the performance of ChatGPT-4.0, a state-of-the-art large language model developed by OpenAI. While not specifically trained on clinical datasets, ChatGPT-4.0 possesses advanced natural language processing capabilities and can simulate human-like reasoning through prompt-based guidance [5]. When provided with structured clinical input, such as sociodemographic details and treatment guidelines, it can generate case-specific treatment recommendations.

This study aims to assess the concordance between ChatGPT-4.0-generated and physician-prescribed smoking cessation treatments by comparing ChatGPT-4.0’s recommendations with real-world clinical decisions made in a specialized smoking cessation clinic. By examining the quality and reliability of AI-generated outputs, the study seeks to contribute to the growing body of literature on the integration of AI tools into patient-specific clinical decision-making processes.

## 2. Methods

### 2.1. Study Design and Setting

This study is a retrospective, descriptive, single-center analysis. It was conducted by reviewing electronic patient records from the Smoking Cessation Clinic at the Şişli Hamidiye Etfal Training and Research Hospital Family Medicine Department, covering the period between 17 December 2024 and 17 January 2025.

### 2.2. Participants and Sampling

Inclusion criteria were as follows:

- Patients aged 18 years or older,
- First-time applicants to the smoking cessation clinic,
- Initiated on pharmacological or behavioral therapy following evaluation.

Exclusion criteria included:

- Pregnant or lactating individuals,
- Patients under 18 years of age,
- Records with missing or inaccurate clinical information,
- Patients not eligible for smoking cessation treatment.

The study population comprised 178 patients (including both initial and follow-up visits) who attended the Smoking Cessation Clinic between 17 December 2024 and 17 January 2025. The sample size was determined based on 96 initial visits, with a power analysis indicating that a minimum of 77 patient records would be required to achieve statistical significance (95% confidence interval, 5% margin of error). Ultimately, 82 patient records meeting the inclusion and exclusion criteria were analyzed. All eligible first-time applicants to the smoking cessation clinic within the defined one-month period were included consecutively.

### 2.3. Data Collection

Patient records were reviewed to extract sociodemographic data (age, gender, occupation, etc.), medical history (chronic diseases, medications, prior smoking cessation attempts), clinical examination findings (vital signs, physical assessment), Fagerström Nicotine Dependence Test (FNDT) scores, daily cigarette consumption, smoking triggers, and physician-prescribed treatments (pharmacological or behavioral therapy). No personally identifiable data were recorded or analyzed.

### 2.4. Clinical Decision Reference Standards

All physician treatment decisions were guided by evidence-based clinical protocols, including the following sources:

- The Turkish Ministry of Health Smoking Cessation Guideline [6],
- The WHO Clinical Treatment Guideline for Tobacco Cessation in Adults [7],
- The Consensus Report on Diagnosis and Treatment of Smoking Cessation by the Tobacco Control Working Group of the Turkish Thoracic Society [8],
- The National Institute for Health and Care Excellence Guideline: Tobacco—Preventing Uptake, Promoting Quitting, and Treating Dependence [9],
- Local clinic protocols developed in accordance with national reimbursement policies and medication availability.

These sources collectively served as the clinical foundation for physician decision-making and were used as an objective benchmark for evaluating the validity of AI-generated treatment recommendations.

### 2.5. AI Evaluation Protocol

ChatGPT-4.0 (OpenAI), a large language model with natural language processing capabilities, was used to generate smoking cessation treatment recommendations for each patient case. Although ChatGPT is a general-purpose AI and not specifically trained on clinical data, it was “primed” with localized contextual information and practice-specific treatment guidelines to simulate clinical decision-making. This included:

- Standard clinical intake forms used at the clinic (also used in data collection for sociodemographic and dependence-related variables),
- National and international treatment algorithms [7,8,9],
- Indications and contraindications for pharmacological treatments derived from national and international clinical algorithms,
- Contextual limitations at the time of the study included:
  ○ Cytisine is available free of charge,
  ○ Varenicline is not available in the country,
  ○ Nicotine patches are available in 16 h forms of 25 mg, 15 mg, and 10 mg,
  ○ Behavioral therapy can be used as monotherapy.

Before the main analysis, a pilot evaluation using 10 anonymized patient records was conducted. This phase aimed to refine the instruction prompts and verify alignment between AI outputs and clinical logic. Adjustments were made to ensure that AI instructions yielded context-appropriate recommendations.

### 2.6. Prompting Protocol

The following standardized prompt was provided to ChatGPT-4.0, before which each structured patient case was presented sequentially:

*‘Based on the structured patient information I will provide, recommend the most appropriate smoking cessation treatment. When making your decisions, rely on the uploaded national and international clinical guidelines as well as treatment indications and contraindications. Take into account the following contextual constraints: cytisine is available free of charge; varenicline is not available in this country; nicotine patches are available in 16-h formulations (25/15/10 mg); behavioral therapy can be used as monotherapy. For each case: (1) Primary treatment recommendation, (2) Possible alternative treatments, and (3) Detailed justification for why these treatments were or were not preferred.’*

### 2.7. Information Quality and Concordance Assessment

For each patient, the ChatGPT-4.0-generated primary treatment, alternative treatment options, and explanatory rationale were reviewed. The clinical accuracy of ChatGPT-4.0’s recommendations and their concordance with the physician’s decision were categorized into three predefined groups:

Perfect Information: The AI recommended the same primary treatment as the physician and provided clinically appropriate, complete justifications for both primary and alternative options.

Useful Information: The AI included the physician’s prescribed treatment among its alternative suggestions and did not recommend any contraindicated treatments.

Inappropriate Information: The AI suggested a treatment that was contraindicated based on the patient’s clinical status or failed to recommend the physician-prescribed treatment.

Concordance was evaluated based on this classification:

Cases classified as Perfect or Useful were considered concordant.

Cases classified as Inappropriate were considered discordant.

To provide contextual clarity to the findings, representative patient cases corresponding to the categories of Perfect, Useful, and Inappropriate information were selected and presented in the study as illustrative examples. These examples were included to illustrate the rationale behind ChatGPT-4.0 decision-making and to demonstrate how it aligned or diverged from physician treatment decisions.

### 2.8. Statistical Analysis

Statistical analyses were performed using IBM SPSS Statistics for Windows, Version 25.0. Descriptive statistics were presented as counts and percentages for categorical variables, and as mean, standard deviation, minimum, and maximum values for numerical variables. Chi-square tests were used to compare proportions in independent groups. Kruskal–Wallis H test was used for comparisons between three independent groups when data did not follow a normal distribution. One-way ANOVA was applied when normal distribution assumptions were met. The Kolmogorov–Smirnov test was used to assess normality. When normal distribution criteria were met, Bonferroni post hoc analysis was performed to analyze group differences. Agreement between physician decisions and AI-generated recommendations was assessed using Cohen’s Kappa statistics. Subgroup analyses were additionally performed by age, gender, regular medication use, and cytisine treatment. Statistical significance was set at *p* < 0.05.

## 3. Results

A total of 82 patient records were analyzed. The mean age was 40.71 ± 12.87 years (range: 19–69), and 40.2% (n = 33) of the participants were female. Sociodemographic data are presented in Table 1.

**Figure 1:**
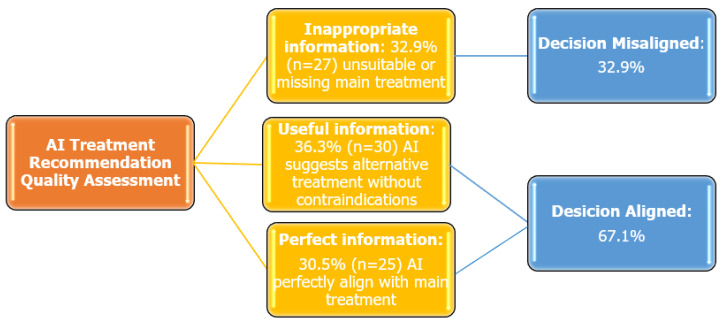
Quality assessment of ChatGPT-4.0 treatment recommendations in smoking cessation.

The overall concordance rate between expert physicians and ChatGPT-4.0-generated treatment recommendations was 67.1%. Based on the quality of ChatGPT-4.0-generated information, the following distribution was observed: Inappropriate Information: 32.9% (n = 27), Useful Information: 36.6% (n = 30), Perfect Information: 30.5% (n = 25) (Figure 1). In addition, Cohen’s Kappa coefficient was calculated to assess agreement between physicians and ChatGPT-4.0, which yielded a value of 0.481 (SE = 0.068, *p* < 0.001), indicating a statistically significant, moderate level of agreement.

Subgroup analyses revealed variability in concordance rates across demographic and clinical characteristics. By age group, the highest agreement was observed in patients aged 40–65 years (κ = 0.47, SE = 0.12, *p* < 0.01), compared with lower values in those aged <40 years (κ = 0.28, SE = 0.11, *p* = 0.04) and >65 years (κ = 0.25, SE = 0.13, *p* = 0.06). Treatment-specific analysis showed that the highest concordance occurred in patients prescribed cytisine (κ = 0.62, SE = 0.09, *p* < 0.001). In contrast, patients with regular medication use exhibited weaker concordance (κ = 0.29, SE = 0.11, *p* = 0.03) compared to those without regular medications (κ = 0.44, SE = 0.10, *p* < 0.01). Agreement was negligible among patients with comorbid diseases (κ = −0.05, SE = 0.12, *p* = 0.64).

**Figure 2:**
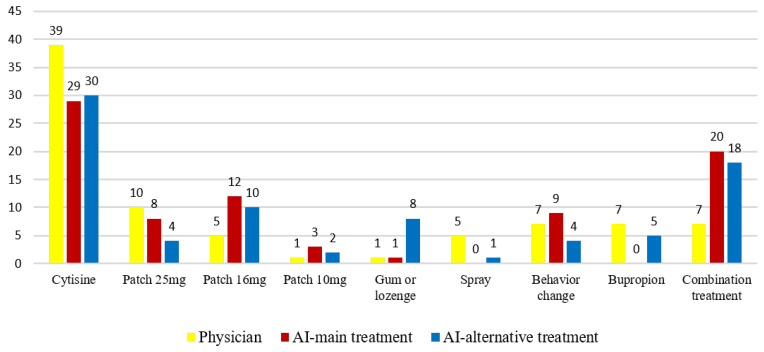
Comparative distribution of treatment preferences between physicians and ChatGPT-4.0 across the study population (n = 82).

Regarding the treatment distribution, physicians most frequently prescribed cytisine therapy (47.6%, n = 39), followed by 25 mg nicotine patch therapy (12.2%, n = 10). ChatGPT-4.0 also most commonly recommended cytisine (35.4%, n = 29), followed by combined nicotine replacement therapy (NRT) (24.4%, n = 20) (Figure 2).

The relationship between the quality of ChatGPT-4.0-generated information and patient characteristics is shown in Table 1. Among cases categorized as Inappropriate Information; 81.5% had a chronic disease, 77.8% were on long-term medication, and 33.3% had abnormal electrocardiogram (ECG) findings.

A significant association was found between information quality and the presence of chronic disease (*p* = 0.021), long-term medication use (*p* = 0.030), and abnormal ECG findings (*p* = 0.011).

The highest proportion of Perfect Information was observed in cytisine therapy recommendations (52.0%), while Inappropriate Information was most commonly associated with nicotine patches, bupropion, and nicotine spray therapies (18.5%).

The relationship between the quality of ChatGPT-4.0-generated information and physician-prescribed treatment modalities was evaluated.

A statistically significant difference was observed in the quality of information provided for patients prescribed cytisine (*p* < 0.001). Within this group, the proportion of “perfect information” was 52%, “useful information” was 73.3%, and “inappropriate information” was 14.8%. According to Bonferroni post hoc analysis, “perfect information” was significantly higher compared to “inappropriate information”. Additionally, “useful information” was also significantly higher than “inappropriate information”.

No statistically significant differences were found in information quality among groups regarding nicotine patches, behavioral modification, bupropion, and combination therapy (*p* = 0.782, *p* = 0.299, *p* = 0.052, and *p* = 0.090, respectively).

To illustrate the discrepancy analysis, representative patient cases with classifications of Perfect, Useful, and Inappropriate information were selected and presented in Table 2. These examples provide detailed insight into ChatGPT-4.0’s reasoning process and help identify common sources of discordance with physician treatment decisions.

## 4. Discussion

This study aimed to evaluate the clinical decision-making performance of ChatGPT-4.0 in the context of smoking cessation treatment by comparing its recommendations with those of expert physicians. The findings demonstrated an overall concordance rate of 67.1%, with a moderate level of agreement according to Cohen’s kappa (κ = 0.41, *p* < 0.001), while 32.9% of ChatGPT-4.0 outputs were classified as clinically inappropriate. These results underscore both the potential and current limitations of ChatGPT-4.0 in replicating human clinical judgment.

Tobacco-related health problems remain one of the leading causes of preventable mortality worldwide. Accordingly, effective smoking cessation interventions are considered a fundamental component of public health policies [14]. While the integration of AI into clinical decision support systems has been widely explored in various medical disciplines [15], to the best of the authors’ knowledge, this is the first study in the literature to directly compare the treatment decisions of ChatGPT-4.0 with those of physicians in a smoking cessation clinic, addressing a significant gap in this field.

The demographic profile of participants reflected a typical primary care smoking cessation cohort, characterized by a wide age range and diverse comorbidities. Notably, AI-physician concordance rates declined significantly among patients with chronic diseases, polypharmacy, or abnormal ECG findings, highlighting the challenges AI models face in complex clinical scenarios. Subgroup analyses further supported these findings: the higher concordance observed in cytisine users may reflect the alignment of AI outputs with guideline-based pharmacotherapy and the relatively uniform treatment protocols for this agent [16]. Similarly, the stronger concordance in middle-aged patients in our cohort could be attributed to more homogeneous clinical characteristics in this age group, although this has not been specifically addressed in prior trials. In contrast, the lack of concordance in patients with comorbidities and polypharmacy highlights the challenges of applying AI models in complex clinical scenarios, consistent with prior evidence that algorithmic performance declines with increasing clinical complexity. These observations collectively indicate that AI recommendations may be more dependable in certain subgroups, but less robust in clinically complex populations. This supports the principle that clinicians should remain the final authority in clinical decision-making and that AI-generated recommendations must be evaluated critically and contextually [17,18].

ChatGPT-4.0 is a general-purpose language model that has not been trained on de-identified health records or integrated clinical datasets. Therefore, its decision-making process relies on probabilistic language modeling rather than the experiential, intuitive reasoning that underpins expert clinical practice [19]. In this study, AI inputs were based on structured data rather than full patient records, which may have further constrained its contextual comprehension.

Although the model was primed with national clinical guidelines and real-world constraints, it failed to adequately consider drug-drug interactions, comorbid conditions, and individual patient risk factors. Prior studies have similarly emphasized the need for training AI systems on real-world, clinically relevant data to improve contextual reasoning and decision accuracy [20,21].

There are several examples in the literature of AI being used in clinical decision support. For instance, an AI model integrated into the DELFOS system has been used to generate personalized treatment recommendations in endometriosis care [22]. In a 2023 systematic review, Haque et al. reported that chatbot-supported AI systems could enhance motivation and assist with patient assessment in smoking cessation, though such systems were largely rule-based and of limited complexity [23]. A deep learning model developed by Google was found to outperform radiologists in pneumonia diagnosis and reportedly reduced clinical workload by up to 30% when incorporated into decision support systems [21,24]. These findings collectively support the expanding role of AI, though its integration into smoking cessation treatment remains in an early developmental stage.

This study also introduced a classification framework to assess the clinical appropriateness of AI-generated recommendations, categorized as Perfect, Useful, or Inappropriate, and analyzed their statistical association with clinical variables such as comorbidity and ECG abnormalities. This approach allows for a broader evaluation of AI performance, encompassing not only outcome concordance but also interpretability, clinical relevance, and safety.

Notably, ChatGPT 4.0 achieved the highest accuracy in cases involving cytisine-based treatments. This finding may be attributed to the widespread use and accessibility of cytisine in the country where the study was conducted. It also suggests that ChatGPT 4.0 may demonstrate enhanced performance when its recommendations align with local pharmacotherapy guidelines. In this context, ChatGPT 4.0 could serve as a supportive tool in managing less complex clinical scenarios or in resource-limited healthcare settings [15,25].

The finding that 32.9% of ChatGPT-4.0 outputs were clinically inappropriate has important safety implications. Inappropriate recommendations may expose patients to substantial risks, such as the use of contraindicated pharmacotherapies in individuals with cardiovascular or neurological comorbidities, unrecognized drug–drug interactions in polypharmacy, or suboptimal treatment selections that may lead to treatment failure and relapse. Particularly in high-risk populations, such as those with cardiovascular disease, epilepsy, or psychiatric conditions, even a single incorrect recommendation could result in serious adverse outcomes. These risks highlight the potential harms of uncritical reliance on AI and underscore the necessity of physician oversight. Thus, ChatGPT-4.0 should be considered adjunctive decision-support tools, with clinicians retaining ultimate responsibility for treatment decisions [26,27].

However, the integration of AI into clinical practice must be undertaken cautiously and incrementally. Regulatory bodies such as the U.S. Food and Drug Administration and the European Union’s Artificial Intelligence Act emphasize the importance of explainability, transparency, and clinical validation prior to AI implementation in healthcare environments [28,29]. Furthermore, ethical concerns, including liability in adverse outcomes, algorithmic bias, and informed consent, require careful consideration [30].

This study has several limitations that should be considered. First, as it was conducted using retrospective data from a single smoking cessation clinic, institutional practice patterns that may influence physician decisions could have impacted the findings, thereby limiting their generalizability. Second, using physician decisions as the reference standard may introduce subjectivity, as clinical decision-making is inherently variable and may not always represent an objective gold standard. Lastly, although the study sample included all eligible patients within the defined period, the relatively small sample size may limit the applicability of the results to broader and more heterogeneous clinical populations. Furthermore, although this study evaluated concordance using ChatGPT-4.0, the findings may not be generalized to all AI-based systems; future research should compare multiple AI platforms (e.g., other large language models or domain-specific AI tools) to provide a more comprehensive evaluation.

## 5. Conclusions

This study offers early insight into the potential of ChatGPT-4.0 to support clinical decision-making in smoking cessation treatment. While the model demonstrated moderate concordance with physician decisions, its performance declined in complex cases—emphasizing the continued need for human oversight and clinical judgment in AI-assisted care.

To improve reliability, future AI models should be trained on diverse, de-identified clinical datasets and designed with hybrid approaches that integrate clinical rules with language-based reasoning. Rather than replacing physicians, ChatGPT-4.0 should be developed as a supportive tool to enhance decision-making, especially in standardized or resource-limited settings. Further prospective, multicenter studies are needed to assess its real-world utility, safety, and acceptance by healthcare providers.

## Figures and Tables

**Table 1:** Association between the clinical quality of ChatGPT-4.0–generated smoking cessation treatment recommendations and patient-specific characteristics.

		Inappropriate Information ^1^	Useful Information ^2^	Perfect Information ^3^	*p*-Value	Post Hoc Comparison
Age * (mean ± SD, min–max)		40.40 ± 14.15 (20–69)	39.20 ± 12.69 (19–63)	42.88 ± 11.82 (19–61)	0.572	
FNBT * (mean ± SD, min–max)		5.92 ± 2.01 (1–9)	6.16 ± 2.29 (1–10)	6.44 ± 2.58 (1–10)	0.724	
Pack-years * (mean ± SD, min–max)		19.18 ± 11.24 (2–42)	23.13 ± 14.39 (5–50)	24.52 ± 12.99 (3–50)	0.306	
Daily cigarette consumption ^ (mean ± SD, min–max)		21.81 ± 12.67 (6–60)	25.26 ± 13.43 (3–80)	23.60 ± 11.14 (4–40)	0.452	
Gender n(%)	Female	11 (40.7)	11 (36.7)	11 (44.0)	0.857	
	Male	16 (59.3)	19 (63.3)	14 (56.0)		
Employment status n(%)	Employed	17 (63.0)	25 (83.3)	16 (64.0)	0.162	
	Unemployed	10 (37.0)	5 (16.7)	9 (36.0)		
Chronic disease n(%)	None	5 (18,5)	16 (53.3)	8 (32.0)	0.021	1 > 2 ª
	Present	22 (81.5)	14 (46.7)	17 (68.0)		
Previous surgery n(%)	None	18 (66.7)	16 (53.3)	10 (40.0)	0.156	
	Present	9 (33.3)	14 (46.7)	15 (60.0)		
Regular medication n(%)	None	6 (22.2)	17 (56.7)	11 (44.0)	0.030	-
	Present	21 (77.8)	13 (43.3)	14 (56.0)		
ECG n(%)	Nsr	18 (66.7)	29 (96.7)	21 (84.0)	0.011	2 > 1 ª
	Other	9 (33.3)	1 (3.3)	4 (16.0)		
Blood pressure n(%)	Regulated	26 (96.3)	29 (96.7)	24 (96.0)	0.991	
	Other	1 (3.7)	1 (3.3)	1 (4.0)		
Mood n(%)	Euthymic	23 (85.2)	30 (100)	21 (84.0)	0.077	
	Other	4 (14.8)	0	4 (16.0)		
Total		27 (100)	30 (100)	25 (100)		

* ANOVA; ^ Kruskall Wallis H; **ª** ANOVA post hoc Bonferroni. Post-hoc comparisons: Group numbers correspond to the following categories: 1 = Inappropriate Information, 2 = Useful Information, 3 = Perfect Information

**Table 2:** Classification framework of ChatGPT-4.0 treatment approaches and information quality across sample smoking cessation cases.

	Patient Information	AI Treatment Recommendation	Physician Treatment	Physician Comment	Decision Concordance
Case 1	24-year-old female 7 pack-years, 20 cigarettes/day. **FNDT:** 5 **Known Medical Conditions (KMC):** Subclinical hypothyroidism **Current Medications (CM):** None **Surgical History (SH):** None **Previous Quit Attempts (PQA):** Two previous trials with nicotine patches, maximum abstinence duration: 1 week. **Psychological Status (PS):** Euthymic, no suicidal ideation **EKG:** Normal sinus rhythm (NSR), **Blood Pressure (BP)**:125/85 mmHg **Smoking Triggers (ST):** Stress, family-related issues, post-meal cravings **Quit Motivation (QM):** Health-related reasons. **Alcohol Use (AU):** None	**Primary Treatment (PT): Cytisine** Justification: Cytisine is currently available free of charge, enhancing patient accessibility. It is an effective medication for moderate nicotine dependence. Subclinical hypothyroidism is not a contraindication for cytisine use. The patient’s previous quit attempts with nicotine patches were unsuccessful, indicating the need for pharmacological support. **Alternative Treatment (AT): Nicotine Patch (15 mg, 16 h release)** Indication: If the patient declines cytisine or experiences side effects, NRT can be initiated. Justification: Effective for reducing nicotine dependence, especially in moderate cases. 25 mg patches may not be necessary initially; a lower dose is preferred. Provides steady nicotine release throughout the day and helps suppress cravings, particularly after meals.	Cytisine	Treatment plan and justifications align completely with the physician’s decision.	Perfect information
Case 44	54-year-old female 42 pack-years, 15 cigarettes/day **FNDT**: 7 **KMC:** Brain neoplasm, Epilepsy, Chronic obstructive pulmonary disease **CM:** Formoterol Tiotropium Levetiracetam **SH:** Right upper lung lobectomy (2023) **PQA**: None **PS**: Euthymic **ECG:** NSR, **BP**: 120/70 mmHg **ST:** Stress **QM:** Health concerns **AU**: None	**PT: Nicotine Patch (15 mg, 16 h release):** Appropriate dose for 15 cigarettes/day and high nicotine dependence. Provides steady nicotine release throughout the day, helping control cravings. **Nicotine Lozenge or Gum (4 mg):** Short-acting support for stress-related triggers. Can be used as needed to manage sudden cravings. Justification: Prevents withdrawal symptoms, which is critical due to underlying COPD and epilepsy. A controlled nicotine supply is a safe and effective strategy in this patient. **AT: Cytisine**—Not Recommended Limited safety data on cytisine use in patients with epilepsy. NRT is considered a safer alternative for this patient. **Contraindicated Treatment: Bupropion** Strictly Not Recommended Lowers seizure threshold and increases the risk of seizures in epilepsy patients. Not suitable for this patient due to high risk of adverse neurological effects.	Nicotine patch 15 mg and gum or lozenge	Treatment plan and justifications align completely with the physician’s decision.	Perfect information
Case 54	54-year-old female 15 pack-years, 4–8 cigarettes/day **FNDT**: 3 **KMC:** Premenopause **CM**: None **SH**: None **PQA**: None **PS:** Dysthymic disorder, no suicidal ideation **ECG:** NSR, **BP**: 115/75 mmHg **ST:** Sadness, post-meal cravings, stress **QM:** Financial reasons **AU:** Occasionally.	**PT: Behavioral Management** (Non-Pharmacological Approach) Justification: Given the patient’s low cigarette consumption (4–8 per day) and low nicotine dependence, non-pharmacological management is generally sufficient. **AT: Pharmacological Support (If Needed): Nicotine Gum, Lozenge (2 mg), or Spray:** Provides short-acting relief for cravings triggered by meals or stress. Justification: Sustained-release options like nicotine patches may not be necessary due to low daily cigarette consumption. **Cytisine: Not recommended** Low dependence level does not warrant its use. **Bupropion: Not recommended** Although the patient has mild dysthymic symptoms, bupropion is unnecessary at this level of dependence.	Nicotine spray	The physician’s treatment was suggested as an alternative by AI, and there were no errors in the AI’s recommendation	Useful Information
Case 45	31-year-old male 11.5 pack-years, 30 cigarettes/day **FNDT**: 8 **KMC:** Aortic and tricuspid valve abnormalities, Acne **CM:** Isotretinoin **SH:** None **PQA:** Tried 6 times, successfully quit for 6 months with bupropion **PS**: Euthymic **ECG:** NSR, **BP**: 120/80 mmHg **ST:** Social environment, alcohol consumption **QM:** Health reasons **AU:** 2–3 beers per week	**PT: Nicotine Patch (25 mg, 16 h release):** Recommended highest dose due to heavy smoking (30 cigarettes/day). Provides continuous nicotine delivery, reducing withdrawal symptoms. **Nicotine Lozenge or Gum (4 mg):** Short-acting relief for cravings, especially in alcohol consumption and social situations. Helps with situational triggers. Justification: NRT is a safe and effective option for patients with high nicotine dependence. **AT: Cytisine** Cytisine is a cost-effective alternative that can be considered. Requires careful monitoring in patients with aortic and tricuspid valve disease, but no strict contraindications exist. **Bupropion Consideration:** May Be Considered with Caution. Patient previously had a successful quit attempt with bupropion. Could be reconsidered, but requires close cardiovascular monitoring. Note: Given the patient’s valvular heart disease, cardiac assessment should be performed before initiating bupropion.	Bupropion	The patient, who had previously experienced a successful outcome with bupropion, was restarted on bupropion treatment after consulting cardiology	Useful Information
Case 65	31-year-old male 20 pack-years, 30 cigarettes/day **FNDT**: 7 **KMC**: None **CM:** None **SH**: None **PQA:** Tried 5 times, but never lasted more than 3 days **PS**: Euthymic **ECG**: NSR, **BP**: 110/70 mmHg **ST:** Stress, coffee, post-meal cravings, social and alcohol-related situations **QM**: Health concerns **AU:** Occasionally (2 times/month, 3–4 whiskey servings).	**PT: Nicotine Patch (25 mg):** Recommended highest dose due to heavy smoking. Provides steady nicotine delivery to reduce withdrawal symptoms. **Nicotine Lozenge or Gum (4 mg):** Short-acting relief for cravings, especially in stressful situations, post-meal cravings, coffee consumption, and social settings. Justification: The patient has attempted quitting five times but was unsuccessful, indicating that NRT can significantly improve cessation success. **AT: Cytisine**: If the patient cannot adhere to nicotine patch treatment, cytisine may be an alternative. A cost-effective option that may help in reducing nicotine dependence. **AT: Bupropion: Can Be Considered** No contraindications in this patient. May aid in stress management and support smoking cessation.	Cytisine	The physician’s treatment was suggested as an alternative by AI, and there were no errors in the AI’s recommendation.	Useful Information
Case 16	69-year-old male. 22 pack-years, 10 cigarettes/day **FNDT:** 1 **KMC:** Benign Prostatic Hyperplasia, Anxiety Disorder **CM:** Tamsulosin Sertraline **SH**: None **PQA**: None **PS**: Euthymic **ECG:** Left axis deviation, first-degree AV block **BP:** 110/70 mmHg. **ST:** Tea and coffee consumption **QT:** Health-related reasons **AU:** None	**PT: Cytisine:** Justification: May be an effective alternative due to the patient’s low nicotine dependence (FNDT = 1). No documented direct interaction between cytisine and sertraline, which the patient is taking for anxiety treatment. **AT: Nicotine Gum (2 mg):** Can be used on demand to suppress sudden cravings in a patient who smokes 10 cigarettes/day. **Nicotine Lozenge (2 mg):** Recommended for specific triggers like tea and coffee consumption.	Behavior change	Pharmacological treatment was not required for low dependence levels. The use of cytisine is not recommended in this age group due to limited clinical evidence. Drug interactions between cytisine and antidepressants are not well-documented.	Inappropriate Information
Case 23	54-year-old male 20 pack-years, 10 cigarettes/day **FNDT**: 6 **KMC:** Recent myocardial infarction (MI) (last week) with two stents placed Mitral regurgitation Tricuspid regurgitation **CM**: Acetylsalicylic acid, Atorvastatin, Ticagrelor, Metoprolol, Perindopril **SH:** None **PQA:** None **PS**: Euthymic **ECG:** Signs of prior MI, atrial enlargement **BP:** 125/85 mmHg **ST:** Coffee, stress, anger, morning craving **QM:** Health-related reasons. **AU:** None	**PT: Nicotine Patch (15 mg):** Provides steady nicotine release to help control cravings in a patient with moderate nicotine dependence. **Nicotine Lozenge or Gum (2 mg):** Recommended for situational cravings, particularly morning smoking urges and coffee consumption. Justification: NRT is considered a safe smoking cessation option post-MI if used with appropriate monitoring of cardiovascular parameters. **Cytisine: Not Recommended**. Caution required in post-MI patients and those with cardiovascular disease. Due to limited clinical data, cytisine is not advised for this patient. **Bupropion: Contraindicated.** Not suitable for patients with cardiovascular risk, especially those with a history of MI. May increase blood pressure and heart rate, further elevating cardiovascular risk.	Behavior change	The 15 mg nicotine patch was deemed inappropriate for the patient with a recent extensive anterior myocardial infarction (MI) history [10,11].	Inappropriate Information
Case 51	28-year-old male 10 pack-years, 20 cigarettes/day. **FNDT**: 4 **KMC:** HIV+. **CM:** Bictegravir/Emtricitabine/Tenofovir Alafenamide. **SH**: None **PQA**: None **PS**: Euthymic **ECG:** NSR **BP:** 100/80 mmHg **ST**: Stress **QM:** Health and financial reasons **AU:** Social drinker	**PT: Nicotine Patch (10–15 mg):** Recommended at a low to moderate dose due to 20 cigarettes/day consumption. Provides continuous nicotine delivery, helping to control cravings and withdrawal symptoms. **Nicotine Lozenge or Gum (2 mg):** Short-acting support for stress-related cravings. Useful for situational triggers (e.g., stress). Justification: NRT is considered safe for HIV+ patients and does not interfere with antiretroviral therapy (ART). Helps manage nicotine withdrawal without compromising HIV treatment. **AT: Cytisine:** An economical option for patients who prefer a non-nicotine pharmacological approach. Limited safety data in HIV+ patients, but no strict contraindications. **Bupropion: Not Recommended** Potential drug interactions with ART medications. May interfere with HIV treatment efficacy. Not preferred in this case due to possible metabolic concerns.	Bupropion	The patient did not want to use NRT. The interaction of cytisine with active ingredients is unknown. There are no known interactions between bupropion and the active ingredients [12].	Inappropriate Information
Case 4	45-year-old female 28 pack-years, 20 cigarette/day. **FNDT**: 8 **KMC**: Hypertension, Insomnia, Major Depressive Disorder, Generalized Anxiety Disorder **CM:** Ramipril, Aripiprazole, Fluoxetine, Quetiapine **SH**: None **PQA**: Attempted quitting independently and with varenicline, but did not use it consistently and relapsed within 10 days. **PS**: Euthymic, no suicidal ideation **ECG:** NSR **BP:** 110/70 mmHg **ST:** Post-meal cravings, coffee consumption **QM:** Health-related reasons **AU:** None	**PT: Cytisine:** Justification: Patient previously attempted varenicline but did not adhere to the full treatment regimen. Given the high nicotine dependence, cytisine may be an effective alternative. Hypertension is well-controlled, making cytisine a safe option. Being available for free improves accessibility and adherence potential. **AT: Nicotine Patch (15 mg):** Can help reduce cravings triggered by meals and coffee consumption. Justification: Provides a steady nicotine level, controlling withdrawal symptoms. Due to high dependence, NRT should be combined with strong behavioral support.	Nicotine patch 25 mg	There is insufficient data on the safety of cytisine use in patients with mental illnesses [13]. There is limited data on drug interactions involving cytisine.	Inappropriate Information
Case 20	28-year-old female 28 pack-years, 20 cigarette/day. **FNDT**: 8 **KMC:** Multiple Sclerosis, Familial Mediterranean Fever, Acne **CM:** Isotretinoin, Glatiramer Acetate, Colchicine **SH**: None **PQA:** Tried at least four times using nicotine gum, patch, spray, and varenicline, but relapsed within 15 days each time. **PS**: Euthymic **ECG:** NSR, **BP:** 100/70 mmHg **ST:** Emotional changes, tea/coffee, social interactions **QM:** Health concerns due to MS **AU:** None	**PT: Cytisine:** Justification: High nicotine dependence necessitates pharmacological support. Can be used cautiously in MS patients, as there are no reported severe neurological side effects with cytisine. However, neurological status should be closely monitored in MS patients. **AT: Nicotine Patch (15 mg):** Provides steady nicotine release to reduce cravings throughout the day. **Nicotine Lozenge or Spray:** Recommended for situational cravings, particularly coffee consumption and social settings. **Bupropion: Not Recommended** May increase the risk of neurological side effects in MS patients, even if rare. Not preferred due to potential worsening of neurological symptoms.	Bupropion	Bupropion treatment was initiated following a neurology consultation. There is insufficient data on the use of cytisine in MS and FMF patients, as well as its potential drug interactions.	Inappropriate Information

## Data Availability

The data that support the findings of this study are not publicly available due to ethical and legal restrictions related to patient confidentiality. However, anonymized data may be made available from the corresponding author upon reasonable request and with permission from the İnstitutional ethics committee.

## Abbreviations

WHO	World Health Organization.
AI	Artificial Intelligence.
